# CICAFAST: comparison of a biological dressing composed of fetal fibroblasts and keratinocytes on a split-thickness skin graft donor site versus a traditional dressing: a randomized controlled trial

**DOI:** 10.1186/s13063-019-3718-4

**Published:** 2019-10-28

**Authors:** Alexandra Poinas, Pierre Perrot, Judith Lorant, Olivier Nerrière, Jean-Michel Nguyen, Soraya Saiagh, Cécile Frenard, Audrey Leduc, Olivier Malard, Florent Espitalier, Franck Duteille, Anne Chiffoleau, Florence Vrignaud, Amir Khammari, Brigitte Dréno

**Affiliations:** 1 Clinical Investigation Centre CIC1413, Nantes INSERM and CHU Nantes, 5, allée de l’île Gloriette, 44093 Nantes Cedex 1, France; 2 Plastic and Reconstructive Surgery Department, Burns Centre, Jean Monnet, CHU Nantes, 30 Boulevard Jean-Monnet, 44093 Nantes Cedex 1, France; 3 CRCINA, INSERM, Université de Nantes, Nantes, France; 4 Cell and Gene Therapy Unit, CHU Nantes, Place Alexis Ricordeau, 44093 Nantes, France; 5 Department of Epidemiology and Biostatistics, CHU Nantes, CRCINA, INSERM 1232, Université de Nantes, Nantes, France; 6 Dermato-oncology Department, CHU Nantes, CRCINA, INSERM 1232, Université de Nantes, Place Alexis Ricordeau, 44093 Nantes, France; 7 Department of ENT and Cervico-facial Surgery, CHU Nantes, 44093 Nantes, France; 8 Sponsor Department, CHU Nantes, 5 Allée de L’île Gloriette, 44093 Nantes Cedex 1, France

**Keywords:** Wound healing, Split-thickness skin graft, Biological dressing, Fetal cells, Randomized clinical trial

## Abstract

**Background:**

Wound repair is one of the most complex biological processes of human life. Allogeneic cell-based engineered skin substitutes provide off-the-shelf temporary wound coverage and act as biologically active dressings, releasing growth factors, cytokines and extracellular matrix components essential for proper wound healing. However, they are susceptible to immune rejection and this is their major weakness.

Thanks to their low immunogenicity and high effectiveness in regeneration, fetal skin cells represent an attractive alternative to the commonly used autologous and allogeneic skin grafts.

**Methods/design:**

We developed a new dressing comprising a collagen matrix seeded with a specific ratio of active fetal fibroblasts and keratinocytes. These produce a variety of healing growth factors and cytokines which will increase the speed of wound healing and induce an immunotolerant state, with a slight inflammatory reaction and a reduction in pain.

The objective of this study is to demonstrate that the use of this biological dressing for wound healing at the split-thickness skin graft (STSG) donor site, reduces the time to healing, decreases other co-morbidities, such as pain, and improves the appearance of the scar.

This investigation will be conducted as part of a randomized study comparing our new biological dressing with a conventional treatment in a single patient, thus avoiding the factors that may influence the healing of a graft donor site.

**Discussion:**

This clinical trial should enable the development of a new strategy for STSG donor-wound healing based on a regenerative dressing. The pain experienced in the first few days of STSG healing is well known due to the exposure of sensory nerve endings. Reducing this pain will also reduce analgesic drug intake and the duration of sick leave.

Our biological dressing will meet the essential need of surgeons to “re-crop” from existing donor sites, e.g., for thermal-burn patients. By accelerating healing, improving the appearance of the scar and reducing pain, we hope to improve the conditions of treatment for skin grafts.

**Trial registration:**

ClinicalTrials.gov, ID: NCT03334656. Registered on 7 November 2017.

## Background

The skin is the largest organ of the human body with numerous complex functions essential for our survival. Its primary function is to act as a protective barrier against the external environment. It can protect against harmful chemicals, ultraviolet radiation and pathogenic organisms, while at the same time producing vitamin D and regulating body temperature and moisture loss. Loss of the integrity of large portions of the skin as a result of injury, illness or burns can lead to major disability or even death; worldwide, burn injuries affect more than 11 million people annually [1]. Wound repair is one of the most complex biological processes of human life. Skin grafting is one of the therapeutic strategies for covering the wound and limiting morbidity.

Currently, the use of an autologous, split-thickness skin graft (STSG) is commonplace as a reconstructive technique for the permanent closure of excised burns or following the excision of benign or malignant tumors. An STSG involves excision, using a dermatome, of the epidermis and part of the dermis, but leaves behind sufficient reticular dermis in the wound bed to enable the skin to regenerate itself. The greatest disadvantage of this technique is that it creates a donor site that is painful during healing, as well as often unsightly scars. The aim of donor-site management is thus to maintain an environment that promotes optimal healing and prevents morbidity, which can include pain and ultimately delayed healing (for review, see [2]).

Theoretically, an appropriate STSG donor-site dressing should reduce patient discomfort, promote rapid healing, decrease pain, prevent hypertrophic scarring and make it possible to “re-crop” from existing donor sites (which can be a high priority in patients with extensive injuries such as thermal injuries). This is the reason for our interest in this clinical application.

It is both most cost-efficient and in the patient’s best interest for one dressing to be applied and remain in situ until healing is achieved. Unfortunately, if an alginate or hydrofiber dressing is left in situ throughout healing, the dressing is likely to dry out and possibly adhere to the wound bed [3]. Furthermore, two systematic reviews of donor-site dressing found no clear evidence to support the choice of any particular dressing [4, 5] except that they must be moist; occlusive or semi-occlusive dressings both fall into this category.

Currently, cell-based, engineered skin substitutes are showing promise for the treatment of acute and chronic wounds such as deep and partial burns, ulcers resistant to conventional therapies and surgical wounds [6–10]. These various engineered tissue formats include allogeneic fibroblasts and keratinocytes in a bovine collagen matrix (OrCel®, Ortec International, Atlanta, GA, USA) [11] or a bilayered living skin equivalent, Apligraf® (Organogenesis, Inc., Canton, MA, USA). Cultured neonatal fibroblasts derived from the human foreskin are combined with bovine type-I collagen to form a neodermis; this is then seeded with cultured neonatal keratinocytes which proliferate and differentiate [12]. Approved in several countries, Apligraf®, as an allograft, has been used in acute wounds such as surgical excision sites and partial-thickness donor sites [12]. Today, the major disadvantages of this biological dressing are its short shelf-life, its cost, its temporary nature [13] and its susceptibility to immune rejection [14, 15]. Previously, it has not been possible to provide both “seed and soil” in the same therapeutic agent, i.e., a complete epidermal layer has never been obtained (for review, see [16]).

Cutaneous wound healing in the early gestation fetus is remarkably different from healing in adults. The most striking features of the fetal wound response are the speed and the absence of obvious scarring, an observation that was first reported more than 30 years ago [17]. Studies in the marsupial embryo, *Monodelphis domestica,* have shown that this fetal regeneration is not due to the moist, sterile environment of the uterus [18]; rather, the nature of this regeneration mechanism could be the result of the difference between the fetal and adult immune responses [19]. Indeed, in utero, the fetal environment demands that the immune system remains tolerant to maternal alloantigens. After birth, the sudden enormous exposure to environmental antigens, many of them derived from intestinal commensal bacteria, calls for rapid change to adapt distinct immune responses so that they are appropriate for early life. However, a study of adult skin grafted onto a fetus, infused with fetal blood and bathed in amniotic fluid found that the wound healed with scar formation [20]. The conclusion of this study is that scarless healing properties are intrinsic to fetal skin and are not primarily the result of the fetal environment, i.e*.,* immune response and/or sterile fluid. In fact, after 24 weeks of gestation, fetal skin repair is histologically indistinguishable from adult skin. This fetal skin-specific phenotype appears to be dictated by quantitative and qualitative alterations in both the inflammatory and regenerative phases compared to normal adult wound healing, due to molecules secreted by fetal skin cells [21]. Consequently, our hypothesis is that these fetal-skin-cell molecules will have a regenerative effect on adult wound healing.

Fetal wound healing features an absence of acute inflammation in association with a low level of pro-inflammatory chemokines, such as interleukin (IL)-8 [22], and a high level of anti-inflammatory cytokines, such as IL-10. This is the opposite of the situation in adult wound healing [23]. In mice lacking IL-10, fetal wounds display substantial inflammatory cell infiltrates and develop scars.

Furthermore, the fetal fibroblasts proliferate at a faster rate [24], with growth and migration rates decreasing with age [25].

In addition, fetal fibroblasts differ from adult in collagen synthesis, with a huge ratio of collagen type III to collagen type I: 3:1, in contrast to 1:3 in adults [26].

In this regard, a novel acellular collagen matrix derived from fetal bovine dermis has recently been designed for dressing partial- and full-thickness wounds (PriMatrix®, from TEI Biosciences Inc., Boston, MA, USA). However, although this scaffold is particularly rich in type-III collagen [27], it contains no cytokines or growth factor crucial for wound healing.

*The novel nature of our regenerative dressing project* is based on four properties of fetal skin cells, which provide an attractive alternative to the keratinocytes and fibroblasts from neonatal foreskin commonly used as skin substitutes:

- Thanks to their low immunogenicity and their immunosuppressive properties they can be used in an allogeneic context without risk of immune rejection
- As a result of the factors they secrete, fetal cells could improve the quality and speed of healing, something which is a constant problem for surgeons. Interestingly, scarless fetal skin healing appears to be largely dependent on the fetal tissue itself and is not reliant on the specific in utero environment [20, 28], giving fetal skin cells great intrinsic potential for wound-healing management
- Pain is decreased by the anti-inflammatory properties of the secreted factors
- Finally, their high proliferation capacity means that from a single skin sample, we can amplify and bank clinical-grade keratinocytes and fibroblasts that can be available to surgeons in 4 days

Furthermore, working on these fetal cells brought us to the conclusion [29] that:

- Keratinocytes and fibroblasts can dramatically inhibit allogeneic peripheral blood mononuclear cell (PBMC) proliferation in a dose-dependent manner, confirming that they induce an immunotolerant state
- This immunosuppressive activity of fetal keratinocytes and fibroblasts mainly relies on IDO (indoleamine 2,3-dioxygenase) activity. Through its ability to locally decrease tryptophan availability, IDO is recognized to exert an immunosuppressive effect on T cells requiring tryptophan to proliferate
- Combining fibroblasts and keratinocytes in co-culture experiments strongly enhances the production of wound-healing growth factors and cytokines (GM-CSF, IL-8, IL-1α, VEGF-A)

These interesting conclusions have led us to build a fetal bioconstruct (biological dressing) that mixes fetal fibroblasts and keratinocytes at a ratio of 1:1 on a type-I collagen matrix (European patent WO 2014 090961A1). This is referred to as CICAFAST, like the trial.

In this trial, the aim of the first application in humans is to test the efficacy of the biological dressing versus a conventional treatment by means of a randomized study. The patient will be their own control, thus avoiding the factors that can influence the healing of a graft donor site. The STSG will be made on the inner part of the thigh at two different sites on the same thigh (upper and lower). The treatment for each site will be chosen by randomization to avoid place bias.

We chose Jelonet® as the conventional dressing since both the Ear, Nose and Throat (ENT) Department and the Burns Center, which are the two investigation centers, use it daily in their practice. Jelonet® (a paraffin-gauze dressing) is the reference treatment for management of the graft donor site. It is essential to differentiate between the graft donor site where a Jelonet® dressing is left in place throughout the duration of healing and the recipient site of the graft or of the wound where the ADAPTIC® (primary dressing made of knitted cellulose acetate fabric and impregnated with a petrolatum emulsion) and UrgoTul® (lipidocolloid dressing) dressings are changed every 24 to 48 h.

This clinical trial should enable the development of a new strategy for STSG donor-site wound healing. The pain of STSG healing during the first days is well known and results from the exposure of sensory nerve endings [30]. Reducing this pain will also reduce the use of analgesic drugs, and sick leave.

Our biological dressing address the essential need for surgeons to “re-crop” from existing donor sites, e.g., for thermal-burn patients. By accelerating healing, improving the appearance of the scar and decreasing pain, we will improve the treatment conditions for skin grafts.

## Methods/design

### Objectives and statistics

#### Objectives

- The aim of this trial is to compare wound healing with CICAFAST versus conventional treatment (Jelonet®) in the treatment of STSG donor sites at Day 8 (D8). Success is defined as a mean healing area of 80% or more at D8, as appraised by a physician observer

The secondary objectives are to:

- Evaluate the concordance between healing at D8 (or D11 and D15) appraised by the investigator observer and the evaluation of healing by another expert physician using photographs

We would have liked to supplement the photos with the addition of histological and immunohistochemical evidence by performing a biopsy on the patient’s scar, but ethically this was not justifiable. To compensate, we therefore use confocal microscopes to examine the quality of the scar.

- Compare the quality of wound healing with CICAFAST versus conventional treatment (Jelonet®) for patients who will be examined using confocal microscopy
- Compare the speed of wound healing with CICAFAST versus conventional treatment (Jelonet®) in the treatment of STSG donor sites
- Evaluate the tolerance of CICAFAST versus conventional treatment (Jelonet®*)* via adverse event (AE) notification
- Compare the pain of wound healing with CICAFAST versus conventional treatment (Jelonet®). The patient will note the number of painful days/surgical wound until complete healing in their patient diary
- Compare the quality of wound healing with CICAFAST versus conventional treatment (Jelonet®). This will be evaluated using two scales: the Observer Scar Assessment Scale (OSAS) and the Patient Scar Assessment Scale (PSAS) [31]

#### Primary objective and sample size

The primary criterion is success as defined by healing at D8 of more than 80% of the surface area treated. The objective is to demonstrate that the CICAFAST dressing is more effective than the control dressing.

Randomization will be applied for all patients who sign the informed consent form and will be performed on the day of surgical intervention. The randomization pertains to the dressing site position for the patient, where H = High (Top); L = Low. Each patient is their own control.

This balanced block randomization is computer-generated. Subjects are randomized into blocks as the allocation progresses, a block being a subgroup of predetermined size within which there is a random allocation of patients. The software used for the randomization is SAS version 9.4. The randomization key is known to the biostatistician and data managers, to make it impossible for the investigator to assign a particular position for the dressings.

The statistical comparison test used for the primary objective is the McNemar test. We tested the hypothesis that the paired difference score is at least 25% in favor of the CICAFAST compared to the control dressing, and the control dressing is never preferred to the CICAFAST dressing. Using McNemar’s *Z* test, two-sided equality, 38 patients will be required for alpha = 5% and beta = 10% (http://powerandsamplesize.com).

#### Secondary objectives

Non-parametric paired tests will be used to compare CICAFAST and the control sites.

The statistical analyses will be performed by Dr. Jean-Michel Nguyen of Nantes University Hospital. Analyses will be performed using the R statistical software, version 3.5. The alpha risk is set at 5% in a bilateral situation.

### Study population

#### Description of the population

Two departments of Nantes University Hospital (France) will be involved in this study: the Department of Plastic and Reconstructive Surgery (Burns Center) and the Department of ENT/Face and Neck Surgery. Due to their patients’ pathologies, both these departments carry out a large number of skin grafts.

In the feasibility study that looked at the PMSI (French Medical Information Systems Program) we determined that the Burns Center and ENT Department carried out 832 STSGs in 2015, easily allowing for the inclusion of 38 patients in this trial.

#### Eligibility criteria and exclusion

The main inclusion criterion is adult patients who need a skin graft (size from 100 to 200 cm^2^ and a thickness of 1.2 mm) following excision surgery. The main exclusion criterion relates to patients with a history of cancers or tumors (except basal cell carcinomas and squamous cell carcinomas). We have preferred not to include patients with a history of cancer since it is the first time that this dressing has been used in humans. The other main non-inclusion criteria relate to:

- Patients suffering from uncontrolled metabolic disease (diabetes, for instance), an untreated psychiatric disorder, severe arteritis of the lower and/or upper limbs, treated with anticoagulants (unless treatment is stopped 7 days prior to surgery), severe venous insufficiency, severe polyneuropathy, or with a known allergy to antibiotics
- Patients with an allergic predisposition or known allergy to bovine collagen or silicone
- Patients receiving corticosteroids, immunosuppressive or cytotoxic agents unless treatment is stopped 4 weeks prior to surgery
- Patients in whom the local anesthetic used in the STSG process at their investigation center is contraindicated.
- Patients with systemic infection (all grades defined by CTCAE Common Terminology Criteria for Adverse Events, V4.03) at the surgery visit will not be included in this trial because of the contraindication to surgery
- Patients who are intolerant to the conventional treatment

### Study design and conduct

#### Study design

This research compares our biological dressing and the reference dressing for STSG donor-site wound healing. The patient will be their own control. This is a single-center, prospective, randomized controlled study comparing the reference treatment, a paraffin-gauze dressing (Jelonet®) to an allogeneic fetal cell-based dressing (CICAFAST).

Two departments of Nantes University Hospital will be involved in this study: the Burns Center and the ENT Surgery Department. As previously mentioned, the pathologies of their patients mean that both these departments carry out a large number of skin grafts.

CICAFAST is produced by the Cell- and Gene-based Therapy Unit (UTCG) of Nantes University Hospital, headed up by Prof. Dréno.

#### CICAFAST dressing

The UTCG has developed a biological dressing based on allogeneic fetal cells seeded on a collagen matrix.

Fetuses come from therapeutic abortions. They are selected according to a gestational age of between 14 and 22 weeks, and they must not have any virus-related or chromosomal abnormalities. The mother’s serology must be negative for: hepatitis B, hepatitis C, HIV, syphilis, HTLV-1 and HTLV.

Clinical-grade keratinocytes and fibroblasts were amplified and banked from a single fetal skin sample. Cell banks were fully characterized and qualified according to the current regulatory recommendations. A GMP (Good Manufacturing Practice) manufacturing process was developed and validated to produce CICAFAST, a new biological dressing combining fetal fibroblasts and keratinocytes in a type-I bovine collagen matrix.

Process and product characterization demonstrated complete compatibility between the fetal cells and the matrix, with homogenous distribution of viable cells and the production of various wound-healing growth factors and cytokines.

The biological dressing composed of fibroblasts and keratinocytes (European patent WO 2014 090961A1) is covered by a hospital exemption for Advanced Therapy Medicinal Products (ATMPs).

#### Conduct of the study (see Fig. 1)

As CICAFAST is being used for the first time in humans, we will include a further patient at least 16 days after CICAFAST is applied to the previous patient. If there is no toxicity once the 10th patient has been included, the Data and Safety Monitoring Committee (DMSC) will be convened to remove this 16-day requirement for the subsequent patients (see the “Adverse event management” section).

**Fig. 1:**
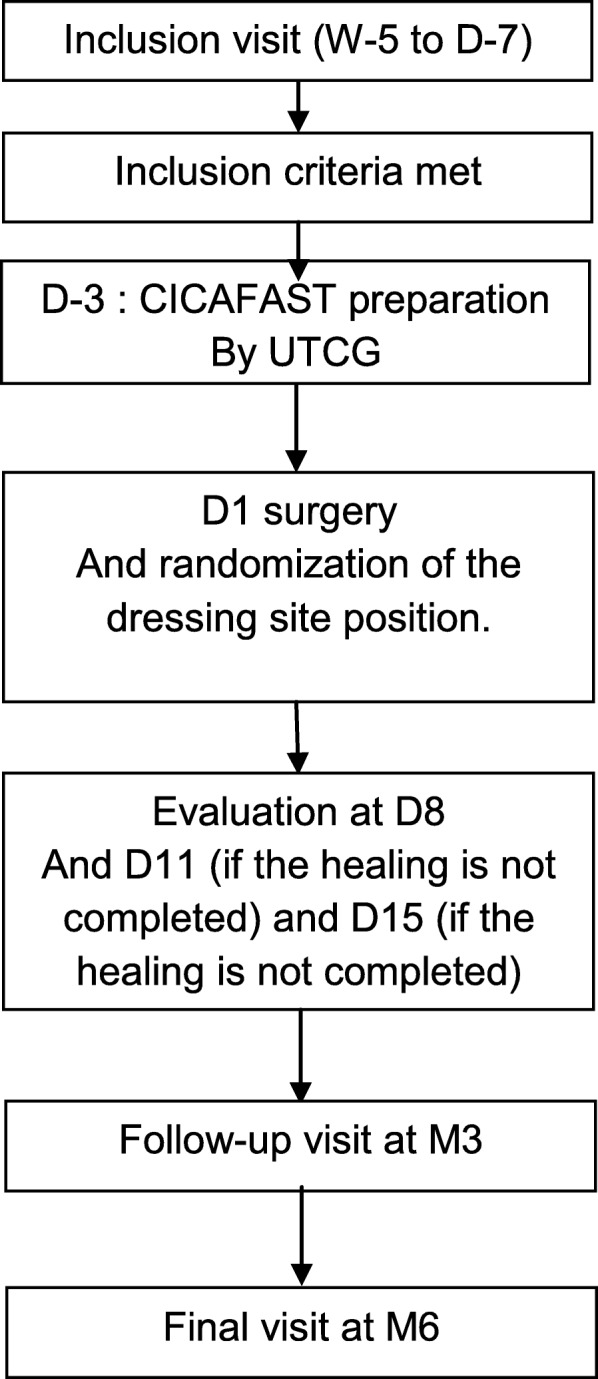
Study diagram

A meeting will be convened for surgeons to ensure that they are proficient with CICAFAST.

Patients who need an STSG of 100 to 200 cm^2^ will be put forward for inclusion in the study. The investigator will then provide the patient with clear and specific information and the patient will sign the informed consent form.

Prior to harvesting, the donor site is prepared under sterile conditions. A local anesthetic agent with or without epinephrine may also be used. The recipient site is measured and the donor site, split into two parts, can be marked to ensure that an appropriate-sized skin graft is harvested, as in current practice. The position of the dressings will be randomized on the eCRF on the operating room by a clinical nurse.

Because CICAFAST is an aqueous dressing, a “silicone barrier” is required. Once CICAFAST is in place, the surgeons will cover it with an ADAPTIC TOUCH® dressing. To hold the CICAFAST dressing in place, the surgeons will use an anti-absorbent dressing (Telfa®) and, a stretch-dressing retention tape (Hypafix® or Nylex®). The wound with the Jelonet® dressing will be treated according to current care: the surgeons place a polyester film over the Jelonet®, making it semi-occlusive; for CICAFAST, an absorbent dressing (Telfa®) is used. As with CICAFAST, stretch tape (Hypafix® or Nylex®) is used to hold the dressing in place.

These stretchable tapes, provided by the sponsor, are held in place by microporous hypoallergenic adhesive tape to secure the dressings.

Both these dressings, one moist, the other semi-occlusive, both fall within the category endorsed by two meta-analyses on “graft donor-site dressings” which have structures that promote better healing and decrease pain. This also removes a bias in this comparison study [4, 5].

The two Jelonet® and CICAFAST dressings are repositionable. They will not be replaced during the patient’s wound healing. However, surgical experience tells us that once the stretch tape has been applied, the CICAFAST dressing will remain in place unless the patient does not want it to.

Also, postoperatively, patients are sometimes kept in hospital for 24 h after skin grafting is performed to ensure that the graft is adequately secured and that the patient’s mobility is limited to prevent shearing forces over the graft site that could dislodge the skin graft. A home health nurse can assess the graft recipient and donor site about 3 days later to ensure that circulation is adequate and that there are no signs of complications. If the patient is in hospital for a prolonged period, such as in the case of a burns patient, then both the recipient and the donor site can be monitored for complications.

The donor site dressing must not be removed until the D8 visit, once agreement has been obtained from a physician.

The patient will be given a diary including two daily Visual Analog Scales (VASs) for the two STSG donor sites, which will allow them to score the wound-healing pain for each donor site every day.

Any drugs deemed necessary for the patient’s well-being and that are not expected to interfere with the assessment of the test dressing, may be administered at the investigator’s discretion.

Patients are permitted to use analgesics to ease their pain (except analgesic gel or ointment), but the patient must make a note of them in their patient’s diary. It should be noted that neither corticosteroid therapy nor inhaled corticosteroids are permitted in this protocol because they can influence wound healing, as can analgesic gel or ointment and all cosmetics. Over-the-counter non-steroidal anti-inflammatory drugs (NSAIDs) are also forbidden.

On D8, the physician will inspect the dressing and may decide that it can be lifted. If they do, the study nurse will lift the two dressings and the physician will then examine the healing.

For CICAFAST, since the dressing is not adhesive, it is easy to evaluate the wound. For the Jelonet® dressing, nurses are used to performing non-traumatic removal and cutting through the Jelonet® on areas that are easily lifted off; the area that can be lifted off corresponds to the re-epithelialized area.

If there is no healing for either site, or if only one of them has achieved healing, two visits can be made, on D11 and D15. As on D8, the physician may decide that the study nurse can lift the dressing and the physician will then evaluate the healing. In addition to the clinical examination, the PSAS (Patient Scar Assessment Scale) and OSAS (Observer Scar Assessment Scale) scores will be recorded at all three visits (see the “Analyses and tests used” section).

Follow-up visits will be performed at 3 and 6 months as per the standard care for patients with STSG. Patients discontinuing the study early should complete the premature withdrawal visit (the same as the M6 visit). The study trial ends at 6 months because it is known that, 6 months after the wound is made, the scar has achieved 80% of the tensile strength of unwounded skin, and it remains at this strength indefinitely [32].

The physician will note any AEs at every visit.

Additional file 1 includes the study schedule in a compressed format.

It should be noted that for compliance of processing, the treatment is administered in hospital. However, the dressing can be removed by the patient, either accidentally or deliberately. This removal AE will be recorded, and the day of removal will also be recorded.

All visit information required by the protocol should be provided in the electronic Case Report Form (eCRF) created by the sponsor (CHU Nantes) and completed by the investigator and his team. The patient will be identified in the eCRF by their initials (the first letter of their first name and family name, supplemented by a number assigned at the time of subject inclusion) and their month and year of birth.

Monitoring visits (to the investigation center and the pharmacy center) will be scheduled regularly by a Clinical Research Associate (CRA) representing the sponsor, and an inspection or audit may take place.

The study will be completed at the end of the participation period of the final subject; in theory, this will be 16 November 2020.

Part or all of the study may be subject to early, permanent or temporary discontinuation by competent authorities, ethics committees or the sponsor.

#### Analyses and tests used

The POSAS is a recent and promising scar assessment tool incorporating both observer and patient scar ratings (it consists of two distinct scales: the OSAS and the PSAS) [31].

The five variables rated by the original version of the OSAS were: thickness, relief, pliability, vascularity and pigmentation. Another item (surface area) was then added in a modified version, after linear regression analysis had shown that the opinion of the observer is most influenced by the dimension of the scar area. In addition, adjectives were inserted alongside the scoring system to better describe each item [33]. The PSAS consists of six items: scar-related pain, itchiness, color, stiffness, thickness and irregularity.

Each POSAS item has a 10-point scoring system, with 1 representing normal skin and 10 the worst imaginable scar or sensation; these items are summed to obtain a total score ranging from 6 to 60 for each scale. In addition to the POSAS score, both observer and patient give their own overall opinion on the appearance of the scar using a 10-point scale.

Both versions of POSAS (original and modified) have been recently validated for application to linear postsurgical scars [33, 34]. The two studies found that both OSAS and PSAS have good internal consistency (Cronbach’s alpha 0.74–0.90) [33, 34].

The modified POSAS is used in both departments.

Furthermore, the two healing sites will be photographed in the operating room after surgery and in the consulting room during the visits at D8, D11, D15, M3 and M6. The resulting pictures will be shown to another physician to evaluate scar healing and quality (80% of re-epithelization for healing and VAS for quality).

At the M3 follow-up visit, in addition to photographs, confocal microscopy (CM) will be used to examine the appearance of the two healing sites compared to a normal control site in the same patient. CM is a non-invasive technique which can be used to obtain in vivo images of skin with a resolution close to that of a histological section. Confocal microscopy enables the examination of the epidermis and the superficial and mid-dermis of the skin. Images up to 8 x 8 mm of the epidermis, the dermo-epidermal junction and the dermis can be used to analyze skin architecture at the different stages in the healing process, as well as scar quality.

### Adverse event management

As CICAFAST is being used for the first time in humans, we will be particularly careful about AEs.

The expected AEs are:

For Jelonet®, no adverse reaction has been registered in the manufacturer’s materiovigilance system/database since 2006 [35]. Although there are no absolute contra-indications to the use of Jelonet®, if the dressing is placed upon a heavily exuding wound, its semi-occlusive nature may cause tissue maceration by preventing the free movement of exudate away from the surface of the wound.

For CICAFAST: four types of AEs could occur:

- Risk of rejection. This AE must be declared to the sponsor, even if the effect does not meet the seriousness criteria
- Due to the traces of antibiotics in the CICAFAST transport medium and despite rinsing prior to application, an allergy risk from these antibiotics must be noted.
- Inadequate placement due to application or tearing
- Quality defect despite the controls by the UTCG of critical and intermediate steps prior to the release of CICAFAST

For Jelonet® and CICAFAST dressing: itching and discomfort and the desire to scratch the healing wound may also occur.

The third AE expected involves removal of the dressing, either accidentally or deliberately. Even if the effect is not serious, it must be declared to the sponsor along with the manner of removal (accidental or deliberate).

In case of infection: patients with grade-1 or -2 infections may be given antibiotics to control the infection. Patients with grade-3 or -4 infections or rejection of the biological dressing (CICAFAST) will have their CICAFAST dressing removed. They will remain in the study, however.

Immunological monitoring will be performed to follow up on CICAFAST rejection by the patient at donor-site level.

#### Data and Safety Monitoring Committee

As CICAFAST is being used for the first time in humans, a Data and Safety Monitoring Committee (DSMC) has been established. The DSMC is an advisory committee responsible for reviewing the safety of a clinical trial for the sponsor and the coordinator of the study. Its members, well versed in the field of clinical trials (pathology and methodology), are not involved in the study. They are appointed for the duration of the study and undertake to take part and observe data confidentiality. The members of the DSMC are appointed jointly by the coordinator and the sponsor. This committee has an odd number of people: a transplant surgeon, a dressings specialist, a cellular therapy specialist and a vigilant.

The DSMC receives the annual safety reports and may be consulted by the monitor if a SUSAR (Suspected Unexpected Serious Adverse Reaction) or SAR (Serious Adverse Reaction) involves a specific analytical problem or in the event of doubt about the risk benefit arising during the course of the study.

### Ethical and regulatory aspects

The clinical study will be conducted in accordance with the French Public Health Code, national and international Good Clinical Practice (GCP) guidelines, and the Declaration of Helsinki, each in the applicable version.

In accordance with French law, the study protocol was submitted and approved by the National Agency for the Safety of Medicines and Health Products (ANSM) on 9 October 2017.

This clinical study was submitted and approved by the Ethical Review Board of Montpellier (Comité de Protection des Personnes Sud-Méditerranée IV) on 9 May 2017. Amendments have been approved by the Ethical Review Board and the regulatory authorities and the updated protocol stands at version 8 as of 9 April 2019.

All submissions/declarations were made by the Sponsor Department of Nantes University Hospital. The Additional file 3 summarizes all the items to address in a clinical trial protocol and that CICAFAST has.

The Ethical Review Board was informed by the sponsor of the first patient inclusion on 16 May 2018.

#### Publication plan

The results of the trial will be published in international dermatology, medical and scientific journals and presented at national and international congresses. The investigators, who will share the entirety of the final trial dataset, will follow the rules and guidelines of the International Committee for Medical Journal Editors (ICMJE) for authorship [36]. The sponsor and the French Ministry of Health, which provided the grant, must be cited in any publication.

## Discussion

The aim of this first human-use trial is to prove both the absence of significant AEs and the efficacy of this new biological dressing.

This trial is the result of multidisciplinary work with the Burns, ENT and Dermatology Departments, as well as with a cell-therapy unit which produced clinical-grade cell banks. A GMP manufacturing process was developed and validated to produce the dressing and conduct regulatory preclinical studies. No sign of toxicity was observed in efficacy/toxicity studies in Wistar rats. In the biodistribution study, clinical monitoring of nude rats showed no signs of acute toxicity of the dressing. The histological conclusion of these studies shows that the use of CICAFAST dressing leads to an improvement in the quality of wound healing compared to the control group treated with the conventional Jelonet® dressing.

Furthermore, a tumorigenicity test was performed by inoculating significant amounts of fetal fibroblasts and keratinocytes in nude mice (10 × 10^6^ cells/animal). Masses were palpable at the administration site (subcutaneous) in all animals following injection. In the light of the regressive character of these masses, two animals were sacrificed on D14 before the masses disappeared completely. At the autopsy, the observations revealed no abnormal organs. Histological analyses concluded that the masses were related to the injection of cells (well-differentiated epithelium) but were not considered as malignant. In addition, it is important to consider that the fibroblasts and keratinocytes (skin cells) were injected subcutaneously. In the absence of a competent local immune system, these cells found an environment conducive to remaining in place for a few weeks before disappearing completely in 24 days. At autopsy of the mice (D84–12 weeks), the observations revealed no abnormal organs or visible tumors. Histological analyses confirmed the absence of tumor. No tumorigenic fetal fibroblasts and keratinocytes were highlighted. No sign of human deoxyribonucleic acid (DNA) was quantified in the organs of the 29 nude mice treated.

As the preclinical studies were favorable, the first clinical trial was written and conducted.

At the time of writing this protocol, in addition to the fact that this is the first administration in humans and that we aimed to check for toxicity, efficacy was also important.

We would have liked a double-blind study in addition to the control and randomization, and that is how we wrote the first version of the protocol. However, the difference in structure, appearance and healing results between the two wounds for the first few patients made it impossible to blind the study either for the patient, the investigator or the expert evaluating the photos of the wound healing. However, to give the protocol maximum scientific level and avoid bias, the following three points have been added:

- To avoid the problem of variable wound depth in ulcers or burns, we chose the graft donor-site model, where the depth is always the same
- To avoid co-morbidities that could affect healing, or to avoid known allocation bias due to the non-blinded way for the patient, each patient acted as their own control in our trial. The two dressings – the reference dressing and our new biological dressing – will be applied at the graft donor site, and the healing of one will be evaluated against the other
- The position of the dressing is randomized so as not to favor one position (and, therefore, one dressing) more than the other

Furthermore, to obtain the best perspective on healing with or without CICAFAST and because it is unethical to perform biopsies on a barely healed wound, in addition to the opinion of the investigator and the patient (P-OSAS), photos will be taken for expert review and the wound will be viewed using reflectance confocal microscopy.

Nevertheless, the lack of blinding is the weak point of our study. CICAFAST is a pilot study, phase 1/2, first-in-human clinical trial. However, if the results of this study are promising, we will then undertake a randomized, double-blind trial where only one dressing per patient would be applied (CICAFAST or Jelonet®) and the difference of the dressing’s release would be evaluated.

## Trial status

The updated protocol stands at version 7 on 20 September 2018.

The first patient was enrolled on 16 May 2018. With an inclusion period of 1 year, it may be possible to enroll the final patient on 16 May 2019 and the study will be ended after their follow-up visit 6 months later.

## Supplementary information

**Additional file 1.** Study schedule.

**Additional file 2.** Informed consent form. The informed consent form given to each patient (French version).

**Additional file 3.** Standard Protocol Items: Recommendations for Interventional Trials (SPIRIT) 2013 Checklist: Recommended items to address in a clinical trial protocol and related documents.

## Data Availability

Data sharing is not applicable to this article as no datasets were generated or analyzed during the current study.

## Abbreviations

AE: Adverse event
CM: Confocal microscopy
CRA: Clinical Research Associate
CTCAE: Common Terminology Criteria for Adverse Event
Day: D
eCRF: Electronic Case Report Form
ENT: Ear, nose and throat
GCP: Good Clinical Practice
GMP: Good Manufacturing Practice
IDO: Indoleamine 2,3-dioxygenase
OSAS: Observer Scar Assessment Scale
PBMC: Peripheral blood mononuclear cell
POSAS: Patient Observer Scar Assessment Scale
PSAS: Patient Scar Assessment Scale
SAR: Serious Adverse Reaction
STSG: Split-thickness skin graft
SUSAR: Suspected Unexpected Serious Adverse Reaction
VAS: Visual Analog Scale
