# Supplementary material for: CICAFAST: comparison of a biological dressing composed of fetal fibroblasts and keratinocytes on a split-thickness skin graft donor site versus a traditional dressing: a randomized controlled trial
Source: Trials. 2019 Oct 28;20:612. doi: 10.1186/s13063-019-3718-4 (PMC6819456; doi:10.1186/s13063-019-3718-4)
Supplement: Supplementary file 1 — Additional file 1. Study schedule. [file 13063_2019_3718_MOESM1_ESM.doc]

| Activities | Inclusion visit (W-5 to D-7) | D-3 CICAFAST preparation | Surgery visit D1 | D8 | If the healing is not completed | D11  If the healing is not completed | D15  If the healing is not completed |  | Visit M3  (± 7 D) | End of the study; visit M6 (± 7 D)  (or withdrawal visit) |
| --- | --- | --- | --- | --- | --- | --- | --- | --- | --- | --- |
| Patient Information | X |  |  |  |  |  |  |  |  |  |
| Patient Consent | X |  |  |  |  |  |  |  |  |  |
| Pregnancy test  For potentially childbearing female | X |  | X |  |  |  |  |  |  |  |
| Immunological monitoring (anti HLA Ig) | X |  |  | X |  |  |  |  | X |  |
| Previous medical history | X |  |  |  |  |  |  |  |  |  |
| Clinical examination | X |  |  | X |  | X | X |  | X | X |
| Treatments | X |  |  | X |  | X | X |  | X | X |
| CICAFAST preparation |  | X |  |  |  |  |  |  |  |  |
| Randomization |  |  | X |  |  |  |  |  |  |  |
| STSG |  |  | X |  |  |  |  |  |  |  |
| JELONET® dressing |  |  | X |  |  |  |  |  |  |  |
| CICAFAST dressing |  |  | X |  |  |  |  |  |  |  |
| Patient’s diary  Given  Recovered |  |  | X | X^a^ |  | X^a^ | X |  |  |  |
| Picture of the wound site |  |  | X | X |  | X | X |  | X | X |
| PSAS scale |  |  |  | X |  | X | X |  | X | X |
| OSAS scale |  |  |  | X |  | X | X |  | X | X |
| Confocal microscopy |  |  |  |  |  |  |  |  | X |  |
| Adverse events |  |  |  | X |  | X | X |  | X | X |

^a^: The patient’s diary is recovered if the two wound healings are completed.
