# Supplementary material for: CICAFAST: comparison of a biological dressing composed of fetal fibroblasts and keratinocytes on a split-thickness skin graft donor site versus a traditional dressing: a randomized controlled trial
Source: Trials. 2019 Oct 28;20:612. doi: 10.1186/s13063-019-3718-4 (PMC6819456; doi:10.1186/s13063-019-3718-4)
Supplement: Supplementary file 2 — Additional file 2. Informed consent form. The informed consent form given to each patient (French version). [file 13063_2019_3718_MOESM2_ESM.doc]

**Informed consent**
