# Supplementary material for: CICAFAST: comparison of a biological dressing composed of fetal fibroblasts and keratinocytes on a split-thickness skin graft donor site versus a traditional dressing: a randomized controlled trial
Source: Trials. 2019 Oct 28;20:612. doi: 10.1186/s13063-019-3718-4 (PMC6819456; doi:10.1186/s13063-019-3718-4)
Supplement: Supplementary file 3 — Additional file 3. Standard Protocol Items: Recommendations for Interventional Trials (SPIRIT) 2013 Checklist: Recommended items to address in a clinical trial protocol and related documents. [file 13063_2019_3718_MOESM3_ESM.doc]

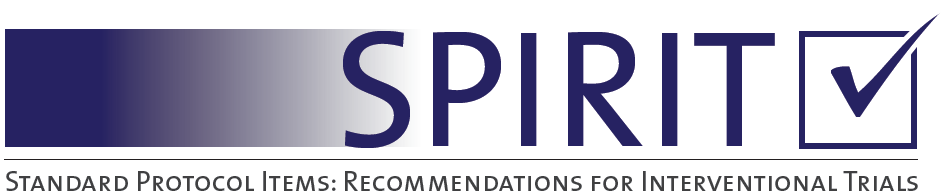


SPIRIT 2013 Checklist: Recommended items to address in a clinical trial protocol and related documents*

| Section/item | Item No | Description | Addressed on page number |
| --- | --- | --- | --- |
| **Administrative information** | | |  |
| Title | 1 | Descriptive title identifying the study design, population, interventions, and, if applicable, trial acronym | **Pg1** in Title of the manuscript |
| Trial registration | 2a | Trial identifier and registry name. If not yet registered, name of intended registry | **Pg 2** in Abstract *- Trial registration* |
|  | 2b | All items from the World Health Organization Trial Registration Data Set (Version1.3.1) | In all the manuscript  **Item1** –**Pg2** Abstract -*Trial registration*  **Item2** **Ditto item 1**  **Item 3** **N/A**  **Item 4** **Pg 25** Funding  **Item 5** **Pg13** Ethical and regulatory aspects  **Item 6** **N/A**  **Item 7** : **Pg1** Corresponding Author **and pg16** Declaration *and Supplementary Material File*  **Item 8** **Pg1** Corresponding Author  **Item 9 Pg16** Declaration *Supplementary Material File*  **Item 10** **Pg 25** Funding  **Item 11** **Pg 7** Study Population in *Description of the population*  **Item 12** **Pg 3 - 6** Background  **Item 13** Study design and conduct in **Pg 8** *Study design* and **Pg9** *Conduct of the study*  **Item 14** **Pg 7** Study Population in *Eligibility criteria and exclusion*  **Item 15** **Pg 8** Study design and conduct in *Study design*  **Item 16** **Pg 13** Ethical and regulatory aspects  **Item 17** **Pg 6** OBJECTIVES AND STATISTICS in *Statistical methods – Primary objective and sample size*  **Item 18** **Pg 13** Ethical and regulatory aspects and **pg14** TRIAL STATUS  **Item19** **Pg 6** Objectives and statistics *Objectives*  **Item 20** **Pg 6** Objectives and statistics *Objectives*  **Item 21** **Pg 13** Ethical and regulatory aspects  **Item 22** **Pg 14** Trial status  **Item 23** **N/A**  **Item 24** **Pg 16** *Declaration and Supplementary Material File in Informed consent to participate*  I |
| Protocol version | 3 | Date and version identifier | **Pg 13** in Ethical and regulatory aspects |
| Funding | 4 | Sources and types of financial, material, and other support | **Pg 25** in Funding |
| Roles and responsibilities | 5a | Names, affiliations, and roles of protocol contributors | **Pg 1** *in the first page of the manuscript*; **pg 7** Study Population in *Description of the population*, **pg 25** in Author’s contribution |
|  | 5b | Name and contact information for the trial sponsor | **Pg13** in Ethical and Regulatory aspects |
|  | 5c | Role of study sponsor and funders, if any, in study design; collection, management, analysis, and interpretation of data; writing of the report; and the decision to submit the report for publication, including whether they will have ultimate authority over any of these activities | **Pg 6,** in Objectives and statistics *Statistical methods*, **Pg13** in *Publication plan* |
|  | 5d | Composition, roles, and responsibilities of the coordinating centre, steering committee, endpoint adjudication committee, data management team, and other individuals or groups overseeing the trial, if applicable (see Item 21a for data monitoring committee) | **Pg 12** Adverse Events managements in *Data and Safety Monitoring Commitee* |
| Introduction |  |  |  |
| Background and rationale | 6a | Description of research question and justification for undertaking the trial, including summary of relevant studies (published and unpublished) examining benefits and harms for each intervention | **Pg 3-6** in Background |
|  | 6b | Explanation for choice of comparators | **Pg 5** in Background |
| Objectives | 7 | Specific objectives or hypotheses | **Pg 5,6** at the end of the BACKGROUND and **pg 6** in Objective and Statistics *in Objectives* |
| Trial design | 8 | Description of trial design including type of trial (eg, parallel group, crossover, factorial, single group), allocation ratio, and framework (eg, superiority, equivalence, noninferiority, exploratory) | **Pg 6-7** Objectives and statistics *in* Statistical methods in *primary objective and sample size*  **Pg 8** in Study Design and conduct in *Study design* |
| Methods: Participants, interventions, and outcomes | | |  |
| Study setting | 9 | Description of study settings (eg, community clinic, academic hospital) and list of countries where data will be collected. Reference to where list of study sites can be obtained | **Pg 7** in Study Population in *Description of the population* **Pg 8** in STUDY DESIGN AND CONDUCT in *Study design* |
| Eligibility criteria | 10 | Inclusion and exclusion criteria for participants. If applicable, eligibility criteria for study centres and individuals who will perform the interventions (eg, surgeons, psychotherapists) | **Pg 7-8** Study population  in *Eligiblity criteria and exclusion* |
| Interventions | 11a | Interventions for each group with sufficient detail to allow replication, including how and when they will be administered | **Pg 9-10** in STUDY DESIGN AND CONDUCT *in Conduct of the study and* **pg 10** *in Analyses and tests used* |
|  | 11b | Criteria for discontinuing or modifying allocated interventions for a given trial participant (eg, drug dose change in response to harms, participant request, or improving/worsening disease) | **Pg12** in ADVERSE EVENT MANAGEMENT in *Data and Safety Monitoring Committee* |
|  | 11c | Strategies to improve adherence to intervention protocols, and any procedures for monitoring adherence (eg, drug tablet return, laboratory tests) | **Pg 9-10** STUDY DESIGN AND CONDUCT in *Conduct of the study* |
|  | 11d | Relevant concomitant care and interventions that are permitted or prohibited during the trial | **Ditto 11c** |
| Outcomes | 12 | Primary, secondary, and other outcomes, including the specific measurement variable (eg, systolic blood pressure), analysis metric (eg, change from baseline, final value, time to event), method of aggregation (eg, median, proportion), and time point for each outcome. Explanation of the clinical relevance of chosen efficacy and harm outcomes is strongly recommended | **Pg 6** in ObjectiveS and Statistics in *Objectives* |
| Participant timeline | 13 | Time schedule of enrolment, interventions (including any run-ins and washouts), assessments, and visits for participants. A schematic diagram is highly recommended (see Figure) | **Pg 7** Study population *Description of the population*, **Pg 9-10** STUDY DESIGN AND CONDUCT *Conduct of the study* and **Figure 1 and Additionnal file 1** |
| Sample size | 14 | Estimated number of participants needed to achieve study objectives and how it was determined, including clinical and statistical assumptions supporting any sample size calculations | **Pg 7** in OBJECTIVES AND STATISTICS *Statistical methods in* Primary objective and sample size |
| Recruitment | 15 | Strategies for achieving adequate participant enrolment to reach target sample size | **Pg 7** Study population in *Description of the population* |
| **Methods: Assignment of interventions (for controlled trials)** | | |  |
| Allocation: |  |  |  |
| Sequence generation | 16a | Method of generating the allocation sequence (eg, computer-generated random numbers), and list of any factors for stratification. To reduce predictability of a random sequence, details of any planned restriction (eg, blocking) should be provided in a separate document that is unavailable to those who enrol participants or assign interventions | **Pg 6,7** Objective and statistics in *Statistical methods* |
| Allocation concealment mechanism | 16b | Mechanism of implementing the allocation sequence (eg, central telephone; sequentially numbered, opaque, sealed envelopes), describing any steps to conceal the sequence until interventions are assigned | **Pg 9 10** STUDY DESIGN AND CONDUCT in *Conduct of the study* |
| Implementation | 16c | Who will generate the allocation sequence, who will enrol participants, and who will assign participants to interventions | **Pg 7** in Study population *Description of the Population,* **pg 9** STUDY DESIGN AND CONDUCT *in conduct of the study* |
| Blinding (masking) | 17a | Who will be blinded after assignment to interventions (eg, trial participants, care providers, outcome assessors, data analysts), and how | **N/A** |
|  | 17b | If blinded, circumstances under which unblinding is permissible, and procedure for revealing a participant’s allocated intervention during the trial | **N/A** |
| **Methods: Data collection, management, and analysis** | | |  |
| Data collection methods | 18a | Plans for assessment and collection of outcome, baseline, and other trial data, including any related processes to promote data quality (eg, duplicate measurements, training of assessors) and a description of study instruments (eg, questionnaires, laboratory tests) along with their reliability and validity, if known. Reference to where data collection forms can be found, if not in the protocol | **Pg 9-10** STUDY DESIGN AND CONDUCT in *Conduct of the study and in* **Pg11** *Analyses and tests used* |
|  | 18b | Plans to promote participant retention and complete follow-up, including list of any outcome data to be collected for participants who discontinue or deviate from intervention protocols | **Pg 9 10** STUDY DESIGN AND CONDUCT in *Conduct of the study* |
| Data management | 19 | Plans for data entry, coding, security, and storage, including any related processes to promote data quality (eg, double data entry; range checks for data values). Reference to where details of data management procedures can be found, if not in the protocol | **Pg 10** STUDY DESIGN AND CONDUCT at the end of *Conduct of the study* |
| Statistical methods | 20a | Statistical methods for analysing primary and secondary outcomes. Reference to where other details of the statistical analysis plan can be found, if not in the protocol | **Pg 6-7** Objective and statistics in *Statistical methods – primary objective an sample size and in* ***Pg 7****secondary objective* |
|  | 20b | Methods for any additional analyses (eg, subgroup and adjusted analyses) | **N/A** |
|  | 20c | Definition of analysis population relating to protocol non-adherence (eg, as randomised analysis), and any statistical methods to handle missing data (eg, multiple imputation) | **Pg 6-7** Objective and statistics in *Statistical methods* |
| **Methods: Monitoring** | | |  |
| Data monitoring | 21a | Composition of data monitoring committee (DMC); summary of its role and reporting structure; statement of whether it is independent from the sponsor and competing interests; and reference to where further details about its charter can be found, if not in the protocol. Alternatively, an explanation of why a DMC is not needed | **Pg 12** ADVERSE EVENT MANAGEMENT in *Data and Safety Monitoring Committee* |
|  | 21b | Description of any interim analyses and stopping guidelines, including who will have access to these interim results and make the final decision to terminate the trial | **Pg 10** STUDY DESIGN AND CONDUCT **at the end** of *Conduct of the Study* |
| Harms | 22 | Plans for collecting, assessing, reporting, and managing solicited and spontaneously reported adverse events and other unintended effects of trial interventions or trial conduct | **Pg12** adverse event management |
| Auditing | 23 | Frequency and procedures for auditing trial conduct, if any, and whether the process will be independent from investigators and the sponsor | **Ditto 21b** |
| Ethics and dissemination | | |  |
| Research ethics approval | 24 | Plans for seeking research ethics committee/institutional review board (REC/IRB) approval | **Pg 13** Ethical and regulatory aspects |
| Protocol amendments | 25 | Plans for communicating important protocol modifications (eg, changes to eligibility criteria, outcomes, analyses) to relevant parties (eg, investigators, REC/IRBs, trial participants, trial registries, journals, regulators) | **Ditto 24** |
| Consent or assent | 26a | Who will obtain informed consent or assent from potential trial participants or authorised surrogates, and how (see Item 32) | **Pg 9** STUDY DESIGN AND CONDUCT in *Conduct of the study* |
|  | 26b | Additional consent provisions for collection and use of participant data and biological specimens in ancillary studies, if applicable | **N/A** |
| Confidentiality | 27 | How personal information about potential and enrolled participants will be collected, shared, and maintained in order to protect confidentiality before, during, and after the trial | **Pg 10** STUDY DESIGN AND CONDUCT in *Conduct of the study* |
| Declaration of interests | 28 | Financial and other competing interests for principal investigators for the overall trial and each study site | **Pg 25** in *Competing interest* |
| Access to data | 29 | Statement of who will have access to the final trial dataset, and disclosure of contractual agreements that limit such access for investigators | **Pg 13** in Ethical and regulatory aspects *in Publication plan* |
| Ancillary and post-trial care | 30 | Provisions, if any, for ancillary and post-trial care, and for compensation to those who suffer harm from trial participation | **N/A** |
| Dissemination policy | 31a | Plans for investigators and sponsor to communicate trial results to participants, healthcare professionals, the public, and other relevant groups (eg, via publication, reporting in results databases, or other data sharing arrangements), including any publication restrictions | **Pg 13** in Ethical and regulatory aspects in *Publication plan* |
|  | 31b | Authorship eligibility guidelines and any intended use of professional writers | **Pg 13** in Ethical and regulatory aspects *in Publication plan* |
|  | 31c | Plans, if any, for granting public access to the full protocol, participant-level dataset, and statistical code | **Pg 16** in Declaration *Supplementary Material File – Informed consent* |
| Appendices |  |  |  |
| Informed consent materials | 32 | Model consent form and other related documentation given to participants and authorised surrogates | **Pg 16** in Declaration *Supplementary Material File – Informed consent* |
| Biological specimens | 33 | Plans for collection, laboratory evaluation, and storage of biological specimens for genetic or molecular analysis in the current trial and for future use in ancillary studies, if applicable | **N/A** |

*It is strongly recommended that this checklist be read in conjunction with the SPIRIT 2013 Explanation & Elaboration for important clarification on the items. Amendments to the protocol should be tracked and dated. The SPIRIT checklist is copyrighted by the SPIRIT Group under the Creative Commons “[Attribution-NonCommercial-NoDerivs 3.0 Unported](http://www.creativecommons.org/licenses/by-nc-nd/3.0/)” license.
